# Recent Advancements in CD47 Signal Transduction Pathways Involved in Vascular Diseases

**DOI:** 10.1155/2020/4749135

**Published:** 2020-07-15

**Authors:** Manman Dou, Ying Chen, Jian Hu, Di Ma, Yingqi Xing

**Affiliations:** Department of Neurology, The First Hospital of Jilin University, Changchun 130021, China

## Abstract

Cardiovascular and cerebrovascular diseases caused by atherosclerosis have a high disability rate and reduce the quality of life of the population. Therefore, understanding the mechanism of atherosclerosis and its control may interfere with the progression of atherosclerosis and thus control the occurrence of diseases closely related to atherosclerosis. TSP-1 is a factor that has been found to have an antiangiogenic effect, and CD47, as the receptor of TSP-1, can participate in the regulation of antiangiogenesis of atherosclerosis. VEGF is an important regulator of angiogenesis, and TSP-1/CD47 can cause VEGF and its downstream expression. Therefore, the TSP-1/CD47/VEGF/VEGFR2 signal may have an important influence on atherosclerosis. In addition, some inflammatory factors, such as IL-1 and NLRP3, can also affect atherosclerosis. This review will be expounded focusing on the pathogenesis and influencing factors of atherosclerosis.

## 1. Introduction

The incidence rate of stroke increased becoming an important issue that affects the quality of life and longevity of human beings. Therefore, it is of vital importance to learn about the causes and preventive measures of stroke. Atherosclerosis is an important factor in stroke formation, and one of the important causes of ischemic stroke caused by atherosclerosis is thrombosis, which is caused by the rupture of vulnerable plaque, which can cause artery-artery embolization [1]. Typical pathological features of vulnerable plaques are a thin fibrous cap, large lipid core, neovascularization, inflammatory infiltration of the plaque envelope and plaque, dilated remodeling, etc. [2]. The mechanisms of atherosclerosis include the theory of lipid infiltration, the theory of endothelial injury, the theory of smooth muscle cell proliferation, the theory of chronic inflammation, and the theory of mononuclear macrophages. Chronic inflammation doctrine is the core element in the development of atherosclerosis in all the theories. A variety of cellular pathways have an important influence on atherosclerosis by influencing plaque vulnerability. The CD47 pathway can generate proinflammatory and anti-inflammatory effects by combining different receptors and ligands.

## 2. Atherosclerosis Formation Mechanism

Atherosclerosis is a chronic inflammatory process, and lipids play an important role in initiating atherosclerosis. Hyperlipidemia activates the congenital and adaptive immune response by activating Toll receptor 2 (TLR-2) and TLR-4 pathways, resulting in the occurrence of inflammation and the expression of atherosclerosis-promoting genes in macrophages and endothelial cells, which can be of huge impact in the process of inducing atherosclerosis [3]. In atherosclerosis, the arterial endothelial wall is injured and low-density lipoproteins (LDL) accumulate in the injured arterial wall. Then, LDL is oxidized to oxidized LDL (ox-LDL), which can lead to a low degree of inflammation within the injured endothelium activating the body's immune response, which plays an important role in the development of atherosclerosis in the initial stage of atherosclerosis. The main inflammatory cells involved in the development of atherosclerosis incorporate monocytes/macrophages, lymphocytes, and so on [4]. Subpopulations of lymphocytes that produce interferon-*γ* (IFN-*γ*) include Th1 cells, CD8^+^ cells, and natural killer cells (NK cells), and they are associated with disease progression while regulatory T cells are associated with a reduced plaque burden [5].

The continued effects of a variety of risk factors including hyperlipidemia, hypertension, diabetes, and other diseases cause endothelial cell damage and the intrusion of LDL into the endothelium. Damaging endothelial tissue and the presence of ox-LDL result in the release of proinflammatory factors, which can increase the cell adhesion molecule (CAM) expression, such as P selectin, vascular cell adhesion molecule 1 (VCAM-1), and intercellular adhesion molecule 1 (ICAM-1) which mediate the adhesion of circulating monocytes and lymphocytes. Monocytes and lymphocytes penetrate the endothelium under the chemokine action of monocyte chemokine-1 (MCP-1) and T cells, respectively, once it sticks to the endothelium. Once they migrate into the endothelium, monocytes differentiate into macrophages due to the macrophage colony stimulating factor (M-CSF), which is considered to be a critical process in the development of atherosclerosis [6].

Macrophages express scavenger receptors such as SR-A, CD36, and lectin-like oxidized LDL receptor-1 (LOX-1) to uptake ox-LDL, leading to lipid accumulation and the formation of foam cell in the injured vascular wall. Furthermore, macrophages promote inflammation and T cell activation through cytokines such as interleukin-1 (IL-1), IL-6, and MCP-1, which can further promote the progress of atherosclerosis. Macrophages can also produce matrix metalloproteinase (MMP) to degrade plaque fiber cap collagen, making the fiber cap thinner and promoting plaque instability [4]. Cytokines secreted by T cells, such as *γ*-interferon and TNF-*α*, stimulate macrophage activation, ox-LDL uptake, and vascular smooth muscle cell (SMCs) migration, promoting atherosclerosis and reducing plaque stability. As this inflammatory process continues, activated lymphocytes, vascular endothelium, and smooth muscle cells secrete fibromediated factors, including various cytokines and growth factors, that promote the proliferation of smooth muscle cells and the proliferation of atherosclerotic plaque substrates. IL-6 is involved in the expression of angiogenesis, c-reactive protein (CRP), and vascular endothelial growth factor (VEGF), which are important factors in atherosclerotic plaque formation [7].

## 3. Innate and Adaptive Immune Response Factors Involved in Atherosclerosis

The deposition of lipoproteins on the intima can trigger an inflammatory response in the arterial wall. The innate immune response is triggered by molecules associated with molecules recognized by the body as non-self, including pathogen-associated molecular patterns (PAMPs) or autologous molecules of damage-associated molecular patterns (DAMP). The adaptive immune response is related to tumor necrosis factor (TNF) produced by T cells or type I interferon (IFN), and some inflammatory factors, such as IL-12, IL-23, and IL-18, can produce proplaque factors and accelerate the progression of atherosclerosis [5]. ox-LDL is the initiating molecule in the process of atherosclerosis, which can be combined with macrophage scavenger receptors such as CD36, SR-A1 and SR-A2, SR-BI, MARCO, LOX-1, and PSOX to boot inflammation and promote atherosclerosis [8]. The effect of CD36 on atherosclerosis has been clearly established. High expression levels of CD36 and LX-1 have been positively associated to the level of IL-1*β*, accelerating the progress of atherosclerosis while SR-A was negatively correlated with IL-8. In addition, important immune regulators essential to inflammation including IL-10, transforming growth factor *β* (TGF-*β*), and lipid mediators which include lipoxins, resolvins, protectants, methylene resins, and prostaglandins are all closely related to the development of atherosclerosis [9].

The formation and development of atherosclerosis is closely related to proinflammatory and anti-inflammatory processes. Anti-inflammatory cytokines play an important role in inhibiting atherosclerosis. Therefore, studying inhibiting factors is crucial for new therapies that can intervene in atherosclerotic disease progress. Anti-inflammatory cytokines associated with CD4^+^ regulatory T cells (Treg) such as interleukin-35 (IL-35), interleukin-10 (IL-10), and transforming growth factor *β* (TGF-*β*) are also involved in disease progression. IL-35 is induced by proinflammatory stimulation and the early development of atherosclerosis and can inhibit endothelial cell activation, while IL-37 and IL-10 also play immunosuppressive roles as anti-inflammatory factors [10].

## 4. Research Progress on the Effect of CD47 Signaling Pathway on the Formation of Atherosclerosis

### 4.1. The Role of CD47 Cytokines in Stroke

CD47 is a cell surface receptor, also known as IAP and as the “don't eat me” signal, and is widely expressed on various cells [11]. Previous studies have shown that the level of CD47 significantly increases in atherosclerotic plaques and patients with cerebrovascular diseases such as a stroke or transient ischemic attack (TIA), and anti-CD47 antibodies can inhibit cell proliferation and aggregation, prevent atherosclerosis, and slow the development of plaques [12]. CD47 can make the immune monitoring mechanism resistant. The signal-regulating protein *α* (SIRP*α*) is an important signaling immunoglobulin that regulates the inflammatory response in macrophages. SIRP*α* (CD172*α*) binds to phosphotyrosine phosphatases SHP-1 and SHP-2 through the cytoplasmic region which is expressed in monocytes/macrophages and neutrophils and can send the “don't eat me” signal. CD47 is a ligand of SIRP*α* and is expressed on the surface of most normal cells that interact with its receptor [13]. When CD47 binds SIRP*α*, the intracellular immune receptors of SIRP*α* are phosphorylated based on tyrosine inhibitory motifs (ITIMs), which subsequently induce the recruitment and activation of phosphotyrosine phosphatases such as SHP-1 and SHP-2. Then, phosphoprotein substrates are dephosphorylated, affecting downstream signaling pathways that transmit the “don't eat me” signal to inhibit phagocytosis of the macrophage [14]. This process is achieved by an increased signal transduction of MAPK (mitogen-activated protein kinase) and increased MerTK (c-mer tyrosine kinase) to promote the release of inflammatory factors such as IL-1*β* [15]. Phagocytosis of macrophages in atherosclerosis leads to aggregation of cells, and cell death and defective cell proliferation eventually lead to the formation of a necrotic core in atherosclerotic plaques, thus accelerating the process of atherosclerosis and increasing plaque vulnerability [16]. Atherosclerosis is an important risk factor for strokes, especially when it appears in the carotid artery or other large arteries.

CD47 also binds to signal-regulating protein *α* (SIRP*α*) to inhibit aggregation of cells [17]. Thrombospondin-1 (TSP-1), a protein released by platelets, can activate CD47, which leads to NO production and inhibition of signal transduction. The CD47-TSP-1 complex causes vascular diseases by promoting the production of reactive oxygen species [11].

### 4.2. Role of CD47 Cytokines in Proliferation

CD47 mediates immune escape, which can prevent macrophage phagocytosis, leading to cell proliferation and aggregation and further to the aggregation of defective cells. Apoptosis is an important aspect of atherosclerosis, and the level of apoptosis varies according to the different stages of atherosclerosis. Studies have shown that the ability to build up cells in atherosclerotic plaques decreases by 20 times in healthy bodies. Dead cells, apoptotic cells, and necrotic debris accumulation are clearly associated with atherosclerosis, necrotic lipid core development, and vulnerable plaques [18]. Secondary necrosis of apoptotic cells and foam cells are considered to be the main cause of necrotic core development and of a gradual development of vulnerable plaques. Under normal physiological conditions, apoptotic cells are quickly cleared by macrophages and other phagocytes through a process called efferocytosis. Recent results suggest that efferocytosis plays an important role in the formation of plaques in humans and animals, which may explain why the necrotic core of cells in plaques accumulates constantly and gradually and aggravates the inflammatory response. Efferocytosis is mediated by macrophages, which balance phagocytic “eat me” signals on apoptotic cells and antiphagocytic “don't eat me” signals such as CD47 molecules. Among the antiphagocytic signaling molecules, CD47 has been identified as a novel therapeutic target for atherosclerosis by promoting the aggregation of cells [19]. Nuclear factor-*κ*B (NF-*κ*B) activation increases the expression of factors such as the tumor necrosis factor *α* (TNF-*α*), and these factors slow down the inhibition of CD47 expression increasing CD47 expression, reducing phagocytosis, and leading to an increase in the necrotic core [20]. The aggregation of cells caused by CD47 not only plays a role in atherosclerosis but also plays a very important role in the development of cancer. Blocking the expression of CD47 and its inhibition with ligands provides a new idea for the treatment of atherosclerosis and cancer [21].

### 4.3. Role of CD47 Cytokines in Inflammation

Atherosclerosis is a chronic, multifactorial disease of the arterial wall characterized by inflammation, oxidative stress, and immune system activation. The regulation of cell aggregation is closely related to inflammation which is a key component of atherosclerosis [18]. Vulnerable plaques are prone to intraplaque bleeding, leading to erythrocyte deposition and hemoglobin release. Red blood cells represent not only an effective mechanism for oxidative stress resistance but also another tool for maintaining immune homeostasis. However, when a strong production of reactive substances occurs, red blood cells can acquire a prooxidant behavior and lose their typical structure and functional characteristics. Under the action of prooxidants, the activation of red blood cells leads to changes, producing more oxidants and free radicals and accelerating the vulnerability of plaques. The expression of CD47 on erythrocytes and the expression of the inhibition of the signal-regulating protein *α* receptor on circulating dendritic cells can inhibit dendritic cell maturation and weaken the adaptive immune response ability of dendritic cells, thereby delaying the progression of atherosclerosis and reducing the vulnerability of atherosclerotic plaques [22]. In the late stage of atherosclerosis, the expression of CD47 in aging erythrocytes can reduce erythrocyte phagocytosis and cause the aggregation of cells accelerating atherosclerosis [15]. It can be seen that CD47 can play a proinflammatory and anti-inflammatory role by interplaying with different ligands in the process of atherosclerosis. In the early stage of atherosclerosis, the proinflammatory mediators play a proinflammatory role over the anti-inflammatory mediators to promote the formation of atherosclerotic plaques. IL-1*β*, IL-6, IL-18, c-reactive protein (CPR), tumor necrosis factor *α* (TNF-*α*), etc., as important proinflammatory factors in the process of atherosclerosis, play an important role in promoting the formation of atherosclerosis. Moreover, the regulation of these factors has also become a target for the treatment of atherosclerosis [23].

### 4.4. CD47 Cytokines' Role in Atherosclerotic Plaque

In atherosclerosis, CD47 expression is significantly elevated and concentrated in the necrotic core of the plaque. Anti-CD47 antibodies can reduce atherosclerosis, which confirms that CD47 is closely related to the formation of atherosclerotic plaques. Some inflammatory factors such as TNF-*α* can promote the expression of CD47 and accelerate the progression of atherosclerosis and plaque formation [12]. A large number of previous studies have shown that CD47 promotes atherosclerosis by expressing the “don't eat me” signal to mediate the endocytosis of macrophages. Recent studies have confirmed the increased plaque formation in CD47-deficient mice and that CD47 has an antiatherosclerosis effect. The loss of CD47 caused the activation of dendritic cells, T cells, and natural killer (NK) cells. CD47^−/−^ can trigger T cell activation, but the activation of T cells will not promote atherosclerosis because T cell activation in CD47^−/−^ mice does not produce more TNF-*α* compared with the wild type. However, CD8^+^ T cells produce more IFN-*γ*, but the proportion of CD4^+^ T cells that can produce IFN-*γ* does not increase. This result suggests CD47 plays a negative role in the process of atherosclerosis. Meanwhile, CD47^−/−^ can activate and increase the expression of CD90^+^ NK cells in CD47-deficient mice, which can produce IFN-*γ* and further promote atherosclerosis [24].

## 5. Effect of CD47 Signaling Pathway on Plaques in Inflammatory Pathways

### 5.1. The Inhibitory Effect of TSP-1/CD47 on Atherosclerotic Plaques

Hypoxia leads to an increased expression of HIF-1-dependent thrombospondin-1 (TSP-1), an extracellular matrix (ECM) glycoprotein secreted and adhered to by neutrophils, monocytes, macrophages, and T cells. Several TSP-1 receptors (CD36, CD47, and integrin *α*_V_*β*_3_) are expressed on various inflammatory cells. TSP-1 in atherosclerosis is involved in multiple processes, including the migration of vascular smooth muscle and the degradation of matrix metalloproteinase 2 (MMP-2), basement membrane, and extracellular matrix, increasing the expression of cell adhesion factors and promoting monocyte recruitment to the vascular wall and migration to the subintima [25]. TSP-1 induces both positive and negative modulations of endothelial cell adhesion, motility, and growth through its interaction with a plethora of cell adhesion receptors, including CD47, CD36, and CD51/CD61, so that TSP-1 can modulate cellular functions including platelet activation and adhesion, leukocyte adhesion, migration, and phagocytosis to have effects on atherosclerosis, thrombosis, angiogenesis, and antiangiogenesis [26]. Studies have shown that the lack of TSP-1 accelerates the maturation of atherosclerotic plaques. A study has shown that in TSP-1-deficient mice, atherosclerosis is accelerated while the lipid core and plaque volume significantly increase [27]. TSP-1 can mediate T cell apoptosis, downregulate the inflammatory response, and resist angiogenesis through CD47, a T cell receptor [28]. The antiangiogenic effect of TSP-1 depends on the ends of their C structure domain, which is shared with CD47 [29]. TSP-1 acts as an angiogenic inhibitor by stimulating endothelial cell apoptosis, inhibiting endothelial cell migration and proliferation, and regulating the bioavailability and activity of the vascular endothelial growth factor. Some studies have found that in the tumor, an increase of TSP-1 and decrease of VEGF lead to a low vascular density in tumor cells, which also indirectly confirms the antivascular properties of TSP-1 [30]. The N-terminal domain of TSP-1 has the activity of promoting angiogenesis [31]. TSP-1 plays a proangiogenic and antiangiogenic role through its C-terminal and N-terminal domains, and CD47 plays a proangiogenic role by combining the C-terminal of TSP-1 [32].

TSP-1 inhibits neovascularization by interacting with VEGF, and CD47 is the only known receptor that can mediate the inhibition of the TSP-1/VEGF signaling pathway [33]. CD47 binding to TSP-1 inhibits VEGFR2 activation and its downstream expression, but does not inhibit VEGF binding to VEGFR2, thereby inhibiting neovascularization [34]. TSP-1/CD47 also inhibit angiogenesis by blocking key angiogenic markers in NO/c-GMP in the endothelium, vascular smooth muscle, platelet, and endothelial cells and inhibit angiogenesis induced by the vascular endothelial growth factor (VEGF) [35]. TSP-1 and its receptor CD47 can also inhibit the activation of NF-*κ*B/AP-1 to limit the proinflammatory effect of the lipopolysaccharide-mediated response and play an anti-inflammatory role through the IL-1*β* pathway [36]. CD47 acts directly with VEGF, while TSP-1 binds to CD47 to regulate the phosphorylation of VEGFR2 and its downstream expression. CD47 can coprecipitate with VEGF, and TSP-1 binding CD47 by the C-terminus can isolate CD47 from VEGFR2 without affecting VEGF binding to VEGFR2, thereby inhibiting downstream AKT activation and activating the functional response of endothelial cells to VEGF to play an antiangiogenic role [37]. VEGF and VEGFR2, as regulators of angiogenesis, are widely expressed in endothelial cells and inflammatory cells such as T cells, and the combination of TSP-1 and CD47 subsequently regulates the expression of VEGF and VEGFR2 [38].

### 5.2. HIF-1/CD47 Signaling Pathway Promotes Atherogenesis

Oxygen plays an important role in atherosclerosis formation, and hypoxia can increase the oxygen-induced factor-1 (HIF-1) expression that exists in macrophages and macrophage-derived foam cells and can promote inflammation, proliferation, and migration of smooth muscle cells, increasing the expression of the vascular endothelial growth factor (VEGF), which can promote neovascularization. VEGF is an important angiogenic factor, which is generally found to be expressed on the surface of endothelial cells, platelets, and other cells. The positive area of VEGF is significantly correlated with the number of intimal vessels and with intracellular hemorrhage in plaques [39]. Hypoxia induces the expression of HIF-1-dependent CD47, which interacts with VEGF to activate antimacrophage phagocytosis, causing hypercytosis, promoting the formation of a plaque necrotic core and accelerating the progression of atherosclerosis. HIF-1, as a regulatory factor of VEGF, can promote angiogenesis and increase plaque vulnerability through the HIF-1/VEGF/VEGFR2 pathway [40]. In addition, HIF-1 is involved in innate and adaptive immune responses, which play an important role in both inflammation and tumors. Hypoxia and chronic inflammation activate the expression of HIF-1 and nuclear factor-*κ*B (NF-*κ*B). In response to inflammation, inhibitory I*κ*B proteins are dissociated from NF-*κ*B allowing its nuclear translocation and activation of tumor-promoting genes including IL-6, cyclooxygenase 2 (COX-2), inducible nitric oxide synthase (NOS2), platelet endothelial cell adhesion molecule-1 (PECAM-1), VEGF, and MMP-9 to promote cancer [41]. In tumors, HIF-1/CD47 can inhibit the antitumoral effect of macrophages through the activation of the CD47 antiligand by SIRP [42]. HIF-1/VEGF levels increase significantly in tumor cells, activating cell proliferation and playing an important role in tumor development [43]. Therefore, the application of HIF-1 antagonists may provide a new direction for the treatment of cancer [44].

## 6. The Influence of CD47 Signaling Pathway on the Formation, Development, and Rupture of Vulnerable Plaques in Inflammatory Pathways

### 6.1. Effect of CD47 Signaling Pathway on Atherosclerosis Formation

CD47 binding with its ligand can promote and inhibit atherosclerosis. The previous view was that in atherosclerosis, an increased expression of CD47 made macrophages resistant to programmed cell removal by expressing the “don't eat me” signal, causing hypercytosis, increasing the necrotic core of the plaque, and promoting the progress of atherosclerosis [45]. CD47 regulates tyrosine kinase receptor activity by interacting with its antireceptor SIRP*α*. CD47 binds the regulating phosphorylation of tyrosine residues in the cytoplasmic domain of SIRP*α* and controls the recruitment of tyrosine phosphatase SHP-2. SIRP*α* binds to VEGF and immunoprecipitates with VEGFR2. CD47 antibody B6H12 downregulates VEGFR2 and SHP-2 phosphorylation [46] and CD47 binds SIRP*α* and TSP-1 to regulate the expression of VEGF and VEGFR2, thereby affecting the progression of atherosclerosis. Recent studies have shown that CD47 plays a role in atherosclerosis not through “cell aggregation” but through the dysregulation and activation of NK cells. CD47 deficiency can accelerate the progression of atherosclerosis, because CD47 deficiency and the loss of CD47-SIRP*α* interaction can cause the activation of T cells and dendritic cells and promote the expression of CD90 on NK cells and the production of IFN-*γ*, both of which can promote the formation of atherosclerosis [24].

### 6.2. CD47 Role in Tumors

CD47, as a “don't eat me” signal, can cause “cell escape,” prevent phagocytosis of macrophages, and cause “cell aggregation.” Besides playing an important role in cell migration and adhesion, angiogenesis, cell proliferation, and apoptosis, CD47 is also widely expressed in a variety of tumors, such as ovarian cancer, bladder cancer, breast cancer, skin cancer, and hematopoietic malignant tumors. SIRP*α* sits on the membranes of myeloid immune cells such as macrophages, dendritic cells, and neutrophils and binds to CD47 with high specificity, triggering antiphagocytic “don't eat me” signals that trigger signaling cascades that lead to myosin dephosphorylation. It is not recruited into the phagocytic synapse subsequently, thereby hindering the phagocytosis of target cells successfully. A high expression of CD47 is considered as an independent prognostic risk factor for tumors [47]. Studies have confirmed that anti-CD47 antibodies can promote phagocytosis of macrophages on tumor cells through different pathways, including the Fc-dependent mechanism, CD47-SIRP*α* axis, and direct induction of apoptosis. This phagocytic process can be realized through the phagocytosis axis of CD47-calcinin, an intracellular protein involved in the dynamic balance of endoplasmic reticulum calcium, which acts as a prephagocytic signal when transported to the cell surface. In addition, it is also possible to activate the adaptive immune response by activating dendritic cells to engulf tumor cells and present tumor antigens to T cells, primarily achieved by inhibiting the CD47-SIRP*α* axis [48]. Studies have confirmed that IgG inhibition of CD47-SIRP*α* interaction in vivo and in vitro significantly enhances tumor cell antibody-dependent cell phagocytosis (ADCP) and cytotoxicity (ADCC) and enhances tumor cell neutrophil phagocytosis [49]. Due to the positive correlation of the CD47-SIRP*α* axis in tumors, the development of CD47-SIRP*α* checkpoint inhibitors and the application of these inhibitors may have some guiding significance for the treatment of tumors. However, since the TSP-1/CD47 pathway has antiangiogenic effects, the lack of CD47 will destroy the antiangiogenic effect mediated by TSP-1 and the NO signal transduction pathway, resulting in increased angiogenesis. Therefore, the side effects of CD47-SIRP*α* checkpoint inhibitors should be considered if they are applied in clinical practice because angiogenesis is an important factor in cancer cell growth [50]. CD47 signaling can also affect T cell activation and death. Activation of CD47 in T cells mainly leads to the suppression of their immune response, while the interaction of CD47 with SIRP*α* or TSP-1 induces proliferation and activation of antigen-specific T cells, thereby improving the immune response to cancer cells [51]. Therefore, in the past years, the study of the “don't eat me” immune checkpoint, namely, the CD47 signaling molecule on macrophages, and the use of inhibitors have provided new ideas for tumor therapy, which may set the basis for a new era of tumor therapy [52].

CD47 is an independent risk factor for tumor prognosis, and inhibiting CD47 can enhance the antitumoral effect. A study shows that blocking CD47 can enhance the antitumor effect of trastuzumab in breast cancer and that the expression of tumor-related cytokine CD47 was inversely correlated with survival in breast cancer patients with HER2^+^. These results may suggest that CD47 may play a protumoral role by promoting cell proliferation and reducing apoptosis [53]. Recent studies have shown that blocking CD47 can inhibit VEGF and enhance antiangiogenesis in NSCLC by enhancing macrophage infiltration and tumor cell destruction. By enhancing the antiangiogenesis of NSCLC, its antitumor effect is also enhanced [54]. These results may indicate that CD47 plays a protumoral role through cell proliferation and decreased apoptosis, which is positively correlated with tumor development.

### 6.3. Role of CD47 Cytokines in Other Diseases

Besides playing an important role in cardiovascular diseases, ischemic strokes caused by atherosclerosis, and tumors, CD47 also plays a significant role in other diseases through different pathways. In intracerebral hemorrhage, CD47 has the ability to accelerate the clearance of hematoma after intracerebral hemorrhage by activating M1-type macrophages and increasing their phagocytosis, significantly reducing brain swelling after hemorrhage, and improving brain tissue damage after acute and chronic intracerebral hemorrhage, which may also become a new target for the treatment of intracerebral hemorrhage [55]. Some studies have also confirmed that in ischemic stroke, when CD47 is deficient, stroke can produce less edema, reduce the infiltration of neutrophils, and cause less secondary brain damage after focal cerebral ischemia, suggesting that CD47 can lead to neurovascular damage to some extent. This is achieved by upregulating neurovascular mediators, such as VEGF and MMP-9. TSP-1/CD47 signal transduction can upregulate the expression of VEGF and MMP-9, thus leading to thrombosis and deterioration and accelerating stroke progression. Therefore, the TSP-1/CD47 pathway may be a new target for the treatment of stroke [56]. In tumors, CD47 can elevate the expression of macrophages and increase their phagocytosis through IL-6, which affects the clearance of tumor cells and has a great impact on the prognosis of tumor cells [57]. By interacting with TSP-1 regulating calcium, nitric oxide (NO), cAMP, and cGMP signal transduction, inhibiting the activation of NK cells and T cell receptor signal transduction, CD47 can become antitumoral. Consequently, CD47 antibody research and the use of its inhibitors can provide new targets for the treatment of various diseases. Interruption of CD47 signal conduction also repairs cell damage caused by ionizing radiation through enhanced autophagy, stem cell self-renewal, and metabolic pathways, to avoid the normal tissue damage [58]. In osteoarthritis, CD47/integrin *α*_V_*β*_3_ also play an important role. In joints, integrin *α*_V_*β*_3_ is produced by synovial cells and expressed in chondrocytes. Proinflammatory genes can be activated after CD47 interacts with integrin *α*_V_*β*_3_, inducing the expression of IL-1, IL-6, CXCL8, IL-8, CCL2, VEGFA, and PTGS2 activation and MMP activation, causing inflammation and the degradation of damaged tissue and promoting disease progression [59]. The CD47 role in the tumor and immune system disease also hints at the positive role of CD47 in the proliferation and growth of defective cells, acting as an important player in disease progression. Therefore, it is not hard to predict that in the process of atherosclerosis, CD47 regulates phagocytosis of macrophages as a kind of “don't eat me” signal and influences plaque formation and vulnerability by regulating other inflammatory cells, such as NK cells, T cell activity to influence the inflammation within the plaque, neovascularization, and cell proliferation.

## 7. Atherosclerosis Formation

In the formation of atherosclerosis, endothelial cells are injured under the action of various risk factors, and LDL keeps accumulating in the vascular wall while being oxidized and modified. Endothelial cells produce a variety of adhesion factors to promote the adhesion of monocytes and lymphocytes in blood vessel walls. After migrating to the intima, monocytes differentiate into macrophages under the action of MCP-1, M-CSF, etc. Macrophages gobble up ox-LDL to form foam cells, which then form lipid streaks and further form early atherosclerotic plaques [60]. Smooth muscle cells migrate to the intima and then begin to proliferate and transform. The fibrous cap is composed of foam cells, inflammatory cells, smooth muscle cells, and an extracellular matrix. HIF-1/CD47/VEGF/VEGFR2 pathways can promote revascularization of plaques. The lipid core, fibrous cap, and angiogenesis form atherosclerotic plaques and angiogenesis increases plaque vulnerability. The TSP-1/CD47/VEGF/VEGFR2 pathway reduces plaque vulnerability by inhibiting angiogenesis in plaques.

## 8. Cytokines as a Therapeutic Target in Atherosclerosis

Several studies have shown that the rate of progression of atherosclerosis of CD47^−/−^ mice was significantly faster than that of wild-type mice [24]. CD47 is widely expressed on various cells as a kind of “don't eat me” signal, causing “cell aggregation,” reducing macrophage phagocytosis, increasing the necrotic core, and finally promoting atherosclerosis. The apoE^−/−^ mice in atherosclerosis induced by angiotensin II (Ang II) reveal that CD47 expression is significantly increased and concentrated in the necrotic core during atherosclerosis development [61]. CD47-blocking antibodies can restrict RhoA-p-MLC signaling pathways and promote phagocytosis of necrotic cells. Therefore, CD47 antibodies have antiatherosclerosis effects [62]. In addition, monoclonal antibodies against IL-1*β* (canakinumab) and methotrexate were also confirmed as efficient for the treatment of atherosclerosis. The CANTOS test proved that inflammatory factor expression such as IL-1*β* and CPR decreases after the action of canakinumab [63]. Inhibiting NLRP3, an activator of IL-1*β*, causes a reduction in the expression and release of inflammatory molecules such as VCAM-1 and ICAM-1 producing atherosclerosis resistance. In addition, IL-6 is a typical inflammatory factor in the process of atherosclerosis, and inhibiting the release of IL-6 and reducing its downstream expression can also have a huge antiatherosclerosis influence [64]. TSP-1 not only plays a role in the inflammatory response but also in immune regulation as an antivascular growth factor. The combination between TSP-1 and CD36 or CD47 or the incubation with anti-CD47 mAb inhibits the differentiation of Th17 cells, inducing CD4^+^ T cell to Treg cell differentiation. To a certain extent, it aggravates the inflammatory response and its symptoms in patients with psoriasis. The development and application of TSP-1 and CD47 inhibitors can provide new therapeutic options for a variety of diseases [28]. VEGF, as a regulator of angiogenesis, can regulate the expression of VEGF in a variety of ways, and it can also regulate inflammation by regulating angiogenesis [65]. HIF-1, as an anoxia inducer, is closely related to anoxia and inflammation. By regulating the expression of macrophages, T cells and other inflammatory cells and inflammatory-related factors such as VEGF can regulate inflammation, tumor proliferation, and migration activities [66]. As mentioned above, TSP-1, HIF-1, CD47, and VEGF all play important roles in inflammation and tumor. Research in this area and the use of antagonists may provide new insights for the treatment of inflammation and tumor. Inflammatory factors such as IL-1, IL-6, MMP, and ICAM are also potential control sites in inflammatory treatment. In conclusion, inflammatory factors and signaling pathways in the process of atherosclerosis can be used as treatment targets and can provide ideas for the prediction of future cardiovascular and cerebrovascular diseases, tumors, and immune system diseases.

## 9. Interaction between CD47 and Other Factors

As an important inflammation regulatory factor, CD47 plays an important role in inflammation, atherosclerosis, and immune diseases. Meanwhile, CD47 also plays different roles by binding to different ligands and receptors. During osteoclast formation, TSP-1 promotes osteoclast formation, angiogenesis, inflammatory response, apoptosis, and fibrosis by binding CD47 and CD36 receptors to inhibit the production and signaling transduction of NO and cGMP signals. CD36, another important regulator of inflammation, has many ligands similar to CD47 [67]. In addition to CD47, CD36 is an important receptor for TSP-1. As mentioned above, the TSP-1/CD47 signaling pathway has an anti-inflammatory effect. Furthermore, TSP-1 can bind to a variety of receptors to perform different functions. For example, TSP-1 plays an anti-inflammatory role by associating with CD47, MMP-9, TGF-*α*, TGF-*β*, and CD36. Moreover, the binding of TSP-1 to CD36 can also have proinflammatory effects [35]. TGF-*β* can also downregulate the expression of the scavenger receptor gene, inhibiting the secretion of CD36, CD68, CD47, and other cytokines and inhibiting the phagocytosis of macrophages, thus regulating the inflammatory response and immune response [68].

TSP-1 presents binding sites for many inflammatory factors including CD47 and CD36. CD47 and CD36 present many similarities, including promoting the expression of inflammatory factors such as IL-1 and NLRP3. In addition, CD47 and CD36 can play a proinflammatory role in many cases [36]. CD36 is better recognized as a scavenger receptor, as it mediates the uptake of ox-LDL by macrophages and the formation of foam cells during arterial atherogenesis to promote atherosclerosis. In the process of atherosclerosis, any factor involved in its formation can contribute to atherosclerosis, including the thin fibrous cap, large lipid core, neovascularization, inflammatory infiltration of the plaque envelope and plaque, and dilated remodeling. CD36 and CD47 not only regulate the inflammatory process in atherosclerosis but also regulate angiogenesis in the process of atherosclerosis [69]. TSP-1/CD36 has been shown to contribute to the sonic hedgehog (SHH) signaling defect in bone marrow-derived angiogenic cells (BMACs) in type 1 diabetic mice, which can disrupt the normal angiogenic process. This gives us a hint that the TSP-1/CD36/SHH signaling pathway can have a role in antineovascularization [70].

As the first antiangiogenic factor discovered, TSP-1 can play an antiangiogenic role through CD36- and CD47-dependent and -independent pathways. Each factor, such as TSP-1, CD36, and CD47, can be combined with different factors to play different roles, such as promoting angiogenesis and antiangiogenesis. Studies have shown that NO is an effective angiogenic factor, while TSP-1 associated with CD47 or CD36 can inhibit the signal transduction of NO, which can inhibit the synthesis of cGMP [71]. In addition to binding CD47 and CD36 to mediate angiogenesis, TSP-1 can also bind directly to VEGF and inhibit the phosphorylation of VEGFR, which is an important angiogenic regulator, thus producing an antiangiogenic effect [72]. *β*1 integrins are also implicated in the antiangiogenic activity of TSP-1 in conjunction with CD36. In conclusion, the TSP-1/CD47 and TSP-1/CD36 signaling pathways play similar roles through many similar pathways, such as antiangiogenesis. Even more, as a scavenger receptor, CD36 also plays different roles in promoting inflammation, antiangiogenesis, promoting angiogenesis, and mediating endothelial cell migration by binding other factors [34]. The similar and different functions of CD47 and CD36 can be seen in Table 1.

In summary, CD36 and CD47 have many similarities regarding their role in inflammation, tumor, and angiogenesis. Furthermore, CD36 and CD47 share common receptors and ligands as inflammatory cytokines. However, the latest research suggests that CD47 as a “don't eat me” signal factor can be combined with TSP-1, SIRP*α*, or other different ligands to play a role in antiangiogenesis. Due to its role in tumor angiogenesis, atherosclerotic plaque, and other pathological changes in the formation process, CD47 is a key player in antiangiogenesis. The study of the CD47 signaling pathway may provide new ideas for regulating the progression of atherosclerosis, delay the progression of atherosclerosis, and thus improve the treatment and intervention of vascular diseases such as stroke, as reducing the incidence of strokes is a top priority.

## 10. The Relationship between Inflammatory Cells and Inflammation

The development of atherosclerosis involves many cells, such as endothelial cells, vascular smooth muscle cells, macrophages, and others. Inflammatory cells exert innate and adaptive immune responses in a variety of similar or different ways. Macrophages, as immune cells, can be activated by a variety of pathways, including the Notch/NF-*κ*B/NLRP3 signaling pathway, and can secrete many inflammatory factors after activation such as TNF-*α* to activate a range of inflammatory responses [73]. CD47 is involved in macrophage-mediated phagocytosis and tumor cell clearance [57]. Macrophage polarization plays an important role in the development of atherosclerosis, inflammation, and tumors. Therefore, regulating macrophage polarization and changing the proportion of macrophage phenotypes in plaques is a new therapeutic approach for atherosclerosis [74]. MIF, a macrophage mobile suppressor, also known as an inflammatory factor, binds to CD74 to upregulate the expression of programmed cell death ligand 1 (PD-L1), which can lead to the immune escape of cancer cells and promote the development of inflammation. In addition, MIF/CD74 can also regulate the expression of inflammatory factors, such as IL-6 and IL-8. Both IL-6 and IL-8 are associated with a cascade of invasion, metastasis, and tumor cell inflammation [75]. Other inflammatory cells, such as T cells, dendritic cells, and natural killer cells, also play an important role in inflammation in various diseases.

## 11. Conclusions

CD47 cytokines play an important role in a variety of diseases, and the use of antagonists and development of inhibitors can be used as a new target of various disease treatments. It can be seen that CD47 causes cell aggregation mainly by affecting macrophage phagocytosis, thus playing a role in cardiovascular diseases, stroke, tumors, and various immune system diseases. Therefore, the TSP-1/CD47 axis and the CD47-SIRP*α* axis play an important role in the occurrence and development of the above-mentioned diseases, and the application of their inhibitors may prove efficient in the treatment of various diseases [76]. Angiogenesis plays an important role in the growth of proliferating cells in atherosclerosis, tumor, and other diseases. TSP-1 binding to CD47 activates vascular growth factor (VEGF) and its receptor (VEGFR2) to regulate angiogenesis while CD47 binding to integrin *α*_V_*β*_3_ promotes neovascularization that facilitates plaque formation, tumor cell growth, and the spread of inflammation. Thus, CD47 can not only cause cell aggregation and participate in the formation and development of various diseases but also bind to different ligands and receptors to promote cell proliferation and inhibit cell proliferation [26].

## Figures and Tables

**Table 1:** Similarities and differences between CD47 and CD36.

Cytokines	Functions	References
CD47	(1) Binds with TSP-1 for antiangiogenesis (2) As a “don't eat me” signal, it blocks the phagocytosis of macrophages and causes cytosis (3) Promoting osteoclast formation, angiogenesis, inflammation, apoptosis, and fibrosis by binding to TSP-1	Kaur et al. [34] Wu et al. [35]
		Kaur et al. [34]
		Koduru et al. [67]

CD36	(1) Anti-inflammatory and antiangiogenesis by binding to TSP-1 (2) Proinflammatory (3) Profibrogenic	Wu et al. [35]
		Rustenhoven et al. [68]
		Koduru et al. [67]

## Abbreviations

LDL: Low-density lipoproteins
NK: Natural killer
VEGF: Vascular endothelial growth factor
HIF: Hypoxia-inducible factors
IL: Interleukin
MMP: Matrix metalloproteinase
TNF: Tumoral necrosis factor
IFN: Interferon
ICAM: Intercellular adhesion molecule
CSF: Colony stimulating factor
SIRP: Signal regulatory protein
NSCLC: Non-small cell lung cancer
SHH: Sonic hedgehog
LOX: Lectin-like oxidized LDL receptor
DAMP: Damage-associated molecular patterns
IAP: Integrin-related protein
TIA: Transient ischemic attack
MAPK: Mitogen-activated protein kinase
MARCO: Macrophage receptor with collagenous structure
ECM: Extracellular matrix
GMP: Guanosine monophosphate
AKT: Protein kinase B
COX: Cyclooxygenase
SIRP: Signal regulatory protein.
